# Prevalence of *mcr-1* in *E*. *coli* from Livestock and Food in Germany, 2010–2015

**DOI:** 10.1371/journal.pone.0159863

**Published:** 2016-07-25

**Authors:** Alexandra Irrgang, Nicole Roschanski, Bernd-Alois Tenhagen, Mirjam Grobbel, Tanja Skladnikiewicz-Ziemer, Katharina Thomas, Uwe Roesler, Annemarie Käsbohrer

**Affiliations:** 1 Department Biological Safety, National Reference Laboratory for Antimicrobial Resistance, Federal Institute for Risk Assessment, Berlin, Germany; 2 Institute for Animal Hygiene and Environmental Health, Freie Universität Berlin, Berlin, Germany; Leibniz-Institute DSMZ, GERMANY

## Abstract

Since the first description of a plasmid-mediated colistin resistance gene (*mcr-1*) in November 2015 multiple reports of *mcr-1* positive isolates indicate a worldwide spread of this newly discovered resistance gene in *Enterobacteriaceae*. Although the occurrence of *mcr-1* positive isolates of livestock, food, environment and human origin is well documented only few systematic studies on the prevalence of *mcr-1* are available yet. Here, comprehensive data on the prevalence of *mcr-1* in German livestock and food isolates are presented. Over 10.600 *E*. *coli* isolates from the national monitoring on zoonotic agents from the years 2010–2015 were screened for phenotypic colistin resistance (MIC value >2 mg/l). Of those, 505 resistant isolates were screened with a newly developed TaqMan-based real-time PCR for the presence of the *mcr-1* gene. In total 402 isolates (79.8% of colistin resistant isolates) harboured the *mcr-1* gene. The prevalence was depending on the food production chain. The highest prevalence was detected in the turkey food chain (10.7%), followed by broilers (5.6%). A low prevalence was determined in pigs, veal calves and laying hens. The *mcr-1* was not detected in beef cattle, beef and dairy products in all years investigated. In conclusion, TaqMan based real-time PCR provides a fast and accurate tool for detection of *mcr-1* gene. The overall detection rate of 3.8% for *mcr*-1 among all *E*. *coli* isolates tested is due to high prevalence of *mcr-1* in poultry production chains. More epidemiological studies of other European countries are urgently needed to assess German prevalence data.

## Introduction

Colistin (polymyxin E) and polymyxin B are polypeptide antibiotics which interact with LPS and phospholipids in the outer cell membrane of gram-negative bacteria. Both polymyxins differ by only one aminoacid with almost equal biological activity. Because of their site effects, polymyxins are rarely used in human medicine but widely used in veterinary medicine. A variety of resistance mechanisms of gram-negative bacteria against colistin and polymyxin B are well-known with a chromosomal localisation [1,2]. In November 2015 the first plasmid-encoded colistin resistance gene *mcr-1* was detected in livestock and raw meat samples as well as human beings in China [3]. Since the first report a multitude of further studies were performed. So far, available reports from Asia [4,5], North Africa [6–8], Europe [9,10], North and South America [11,12] showed a global spread of this gene. To date *mcr-1* is mostly detected in *E*. *coli*, but the occurrence in *Salmonella*, *Shigella*, *Klebsiella*, *Vibrio* and *Enterobacter* was also sporadically reported [13–16]. The *mcr-1* positive microorganisms have been isolated from various sources–like the environment [17], livestock [18] and food [19,20] but also from infected human patients [21,22] as well as asymptomatic human carriers including international travellers [23]. In consideration of trading with food producing animals and retail meat, spread of *mcr-1* mediated colistin resistance between countries has taken place as shown by Grami et al. (2016) [6].

Reports describing *E*. *coli* isolates carrying a plasmid-encoded *mcr-1* gene in combination with carbapenemases are most concerning [24–26]. In human medicine, nowadays colistin is one of the last therapeutic options for the treatment of infections caused by carbapenemase producing bacteria. Therefore, the current situation has to be assessed critically. On the other hand, in veterinary medicine, colistin has been widely used for decades for the treatment of diarrhoea in food-producing animals, especially pigs and poultry. This indicates that the worldwide spread of the plasmid-encoded colistin resistance gene *mcr-1* reflects a major topic at the interface between human and animal health. However, sales data indicate a reduction by 15.8% of colistin-sales to veterinarians in Germany between 2011 and 2014 from 127 to 107 tons of substance per year [27].

More comprehensive data on the prevalence of plasmid-mediated colistin resistance in different matrices are urgently needed to assess the impact of colistin usage in veterinary medicine on the development of resistance situation in bacteria causing infections in human patients. A first systematic screening addressing the prevalence of the *mcr-1* gene in *E*. *coli* from livestock was performed in France [9] resulting in a prevalence of 0.5% in pigs, 1.8% in broilers and 5.9% in turkey. For Germany, until now only single isolates are reported [25]. To get more detailed information about the spread of *mcr-1* in Germany, this study describes the systematic screening of *E*. *coli* isolates from German livestock and food samples derived from the German monitoring program on antimicrobial resistance in zoonotic agents during the years 2010–2015. A TaqMan based real-time PCR assay was developed as an efficient and rapid screening method for the investigation of high sample numbers and its functionality was proven in different laboratory-settings.

## Materials and Methods

### Establishment and validation of the TaqMan-based real-time assay

Reference sequence data of the *mcr-1* reference gene was derived from the GenBank web site (KP347127; http://www.ncbi.nlm.nih.gov/genbank/) and the primer and the probe design was performed using the online PrimerQuest Tool (http://eu.idtdna.com/primerquest/home/index):

RT-mcr-1_F—TGGCGTTCAGCAGTCATTAT;

RT-mcr-1_R—AGCTTACCCACCGAGTAGAT;

RT-mcr-1_Probe—Cy5-AGTTTCTTTCGCGTGCATAAGCCG-BBQ-650 (biomers.net GmbH; Ulm, Germany).

DNA preparation was done as previously described [28]. The TaqMan PCR amplifications were performed in 25 μL reactions containing 12.5 μL ABsolute qPCR Mix (Thermo Scientific, St. Leon Roth, Germany), 1 μL of RT-mcr-1_F and RT-mcr-1_R (10 pmol), 0.2 μL of the TaqMan probe (10 pmol), 9.3 μL of sterile water and 1μL of DNA preparation.

To determine the optimal real-time PCR conditions and to confirm the specificity of the assay, a set of ten positive control strains (P1-10), known to contain the *mcr-1* gene and ten negative control strains (N1-10) were chosen and tested in triplicates. The obligatory “no template control” (NTC) was part of every single real-time run. To proof the functionality of the assay in variable settings the real-time runs were performed analogous in two different laboratories using either the LightCycler 480II (Roche Diagnostics GmbH, Mannheim, Germany) or the CFX96 (Bio Rad Laboratories GmbH, Munich, Germany). The PCR conditions were used as follows: To achieve a maximum of polymerase activity a preliminary heating step at 95°C for 15 min was necessary. This was followed by 30 cycles of 95°C for 15 sec and 60°C for 1 min. Fluorescence signals were detected in the channel 618–660 nm (Lightcycler480II, Roche) or channel 4: Cy5 (CFX96, Bio-Rad). Following each run, a cycle threshold (Ct) was calculated by determining the signal strength at which the fluorescence exceeded a threshold limit. This value was manually set at LightCylcer 480II and samples possessing a signal above this value were assessed as positive.

### Conventional PCR

Negative control strains were obtained by a pre-screening of phenotypically colistin resistant isolates from the German national monitoring on zoonotic agents with conventional PCR using primers described by Liu et al. (2015) with the following conditions: annealing at 54°C for 30 sec and elongation at 72°C for 30 sec [3]. To prove the real-time results of the colistin-resistant isolates from the German monitoring program, conventional PCR followed by the subsequent sequencing of the PCR products was carried out on a random set of isolates. For this, the primers described by Falgenhauer et al. (2016) were used [25]. Amplified PCR fragments were purified for sequencing using the innuPREP PCRpure Kit (Analytik Jena, Jena, Germany). Sequencing of the PCR products was conducted by an external service provider (LGC Genomics, Berlin, Germany). The obtained sequences were analysed using the program ‘‘SeqMan Pro” of the Lasergene10 Core Suite (DNASTAR, Inc., Madison, USA).

### Bacterial control strains

The *mcr-1* positive and negative controls, used for the establishment of the real-time PCR assay, were derived from different sources. Along with the positive control DNA, provided by the European Union Reference Laboratory for Antimicrobial Resistance, Lyngby, Denmark, the German reference strain 15-AB00353 from the German Reference Laboratory for Antimicrobial Resistance, Berlin, Germany, and the already published isolate R253 were included [25]. The remaining seven isolates originated from the strain collection of the Institute of Animal Hygiene and Environmental Health (Free University (FU-) Berlin, Germany). The presence of the *mcr-1* gene was confirmed by conventional PCR and subsequent sequencing of the PCR-product. The ten *E*. *coli* isolates serving as negative controls originated from the German Reference Laboratory for Antimicrobial Resistance and were previously tested in a conventional PCR format (see above). The final validation and reliability of the assay was tested in two different laboratory settings as described above, using a defined set of 96 *E*. *coli* isolates from the German national monitoring in zoonotic agents.

### Investigated *E*. *coli* strains from the German monitoring program on antimicrobial resistance in zoonotic agents

A total of 505 phenotypically colistin resistant *E*. *coli* isolates from the German monitoring program on antimicrobial resistance in zoonotic agents during the years 2010 and 2015 were included in this study. This national monitoring program is in concordance with Directive 2003/99/EG and Decision 2013/652/EU of the European Union. It includes resistance determination in commensal *E*. *coli* isolated from faecal and food samples of animal origin [29]. Minimal inhibitory concentrations (MIC) for several antimicrobials were determined by broth microdilution method following CLSI-guidelines (CLSI MK07-A10, 2013/652/EU) and using SENSITITRE MIC plates (TREK Diagnostic Systems, Thermo Scientific). Since 2010, colistin concentrations covering the epidemiological cut-off value defined by EUCAST (MIC≥4mg/l) were implemented in the test panel. In total, 10,609 *E*. *coli* isolates were tested, resulting in 505 *E*. *coli* isolates designated as resistant to colistin. These isolates were screened for the presence of the *mcr-1* gene.

## Results

The validation of the here described TaqMan PCR assay using ten *mcr-1* positive (P1–10), ten *mcr*-1 negative (N1-10) *E*. *coli* isolates and a no template control (NTC) was successful. C_t_ values measured in both laboratories in three technical replicates are given in Table 1. All of the positive control strains were definitely detected on the LightCycler 480II (Roche) as well as on the CFX96 (Bio-Rad). Variations between the three replicates were small (standard deviation range between 0 and 0.7). In case of the LightCycler the detected fluorescence signals crossed the threshold line on an average of 15 completed cycles. In case of the CFX96 (Bio-Rad), the detected C_t_ values were slightly higher (~18). False positive signals were neither detected in any of the ten tested negative control strains nor in the NTC.

**Table 1:** Validation of the real-time PCR assay. For the assay validation ten positive (P1-10) as well as ten negative (N1-10) control strains were used. The runs were performed in three technical replicates and the mean C_t_ values as well as the resultant (standard deviation) are indicated.

Sample	Roche LightCycler 480 II	BioRad CFX 96
**NTC**	none	none
**N1 to N10**	none	none
**P1**	13.80 (0.29)	17.36 (0)
**P2**	18.83 (0.67)	21.10 (0.33)
**P3**	14.92 (0.17)	17.95 (0.70)
**P4**	15.81 (0.09)	18.16 (0.24)
**P5**	16.51 (0.07)	19.78 (0.24)
**P6**	15.52 (0.47)	18.25 (0.31)
**P7**	13.61 (0.23)	18.42 (0.08)
**P8**	14.23 (0.36)	18.09 (0.12)
**P9**	15.08 (0.19)	18.21 (0.17)
**P10**	15.61 (0.30)	19.08 (0.24)
**Total (P1-10)**	**15.29 (1.48)**	**18.64 (1.06)**

NTC = No Template Control

In the final validation, DNA-preparations from 96 phenotypically colistin resistant *E*. *coli* isolates were tested in two independent laboratories using the two different real-time cycler systems. The previously established PCR conditions turned out to be stable in both laboratories. In case of the *mcr*-1 positive isolates mean C_t_ values of 13 (Roche LightCycler) vs. 15 (BioRad CFX96) were determined. The classification of *mcr-1* positive as well as negative isolates in both laboratories matched 100%. Finally, ten of the positive tested DNAs were randomly selected and the *mcr-1* gene was amplified in a conventional PCR format and confirmed via sequencing with 100% identity to the *mcr-1* gene described by Liu et al. (2015) [3].

Out of 10,609 commensal *E*.*coli* isolates, collected during the years 2010–2015, 505 isolates showed MIC-values >2 mg/l for colistin (4.8%). In 402 of these phenotypically colistin resistant isolates (79.6%) the *mcr-1* gene was detected by PCR. Based on the assumption that isolates with an MIC of < = 2mg/l will not harbour *mcr-1*, an overall *mcr-1* prevalence of 3.8% among all the 10,609 *E*. *coli* isolates was determined.

Huge differences in the prevalence of colistin-resistance and the *mcr-1* between the different animal origins were detected (Table 2). The highest prevalence of *mcr-1* was found in turkeys (animal) with an overall prevalence of 11.8%. The observed variation over the years reflects that different matrices were sampled in the different years. Table 2 illustrates that the highest *mcr-1* prevalence was observed in isolates from faecal samples taken at farm level, whereas the prevalence was continuously lower in isolates from caecal samples at slaughter and meat samples at retail. While there was no clear trend for the prevalence in faecal samples over time, the detection rate of *mcr-1* in *E*. *coli* isolated from turkey caeca samples decreased from 9% in 2012 to 3.8% in 2014 (Table 2). This decreasing trend is also observed in turkey meat with a prevalence close to 10% in the years 2010 and 2012 and lower (5.4%) in 2014. The proportion of the *mcr-1* gene carriers among colistin resistant isolates was very high and even increasing over the time up to 94.9% in turkey livestock samples in 2014 (Table 2).

**Table 2:** Prevalence of *mcr-1* in German livestock and food samples 2010–2015.

Sample origin	Year	Matrices	No. of isolates investigated	No. of colistin resistant isolates	No. of *mcr-1* positive isolates	Prevalence *mcr-1* (in %)	Prevalence colistin resistance	Proportion *mcr-1* positive *E*. *coli* among colistin resistant isolates
Laying hens	2010		802	9	0	0.0	1.1	0.0
	2011		642	13	2	0.3	2.0	15.4
	2014		351	2	1	0.3	0.6	50.0
Eggs	2014		90	2	0	0.0	2.2	0.0
Broilers	**2010**	at farm, faeces	147	8	8	**5.4**	5.4	100.0
	**2011**	at farm, faeces	246	18	17	**6.9**	7.3	94.4
	**2013**	all	667	57	52	**7.8**	8.5	91.2
		at farm, faeces	161	4	4	2.5	2.5	100.0
		at slaughter, caeca	273	27	24	8.8	9.9	88.9
		at slaughter, carcass	233	26	24	10.3	11.2	92.3
	**2014**	all	414	25	22	**5.3**	6.0	88.0
		at farm, faeces	184	9	8	4.3	4.9	88.9
		at slaughter, caeca	230	16	14	6.1	7.0	87.5
Chicken meat	2011		172	16	14	8.1	9.3	87.5
	2013		207	13	10	4.8	6.3	76.9
	2014		201	2	1	0.5	1.0	50.0
Turkey	**2010**	all	381	46	39	**10.2**	12.1	84.8
		at farm, faeces	107	14	14	13.1	13.1	100.0
		at slaughter, caeca	274	32	25	9.1	11.7	78.1
	**2011**	at farm, faeces	184	37	33	**17.9**	20.1	89.2
	**2012**	all	537	68	63	**11.7**	12.7	92.6
		at farm, faeces	205	36	33	16.1	17.6	91.7
		at slaughter, caeca	332	32	30	9.0	9.6	93.8
	**2014**	all	357	39	37	**10.4**	10.9	94.9
		at farm, faeces	173	30	30	17.3	17.3	100.0
		at slaughter, caeca	184	9	7	3.8	4.9	77.8
Turkey meat	2010		181	19	17	9.4	10.5	89.5
	2012		307	32	30	9.8	10.4	93.8
	2014		188	11	10	5.3	5.9	90.9
Beef cattle	2011	at farm, faeces	909	6	0	0.0	0.7	0.0
	2013	faeces and colon content	526	0	0	0.0	0.0	-
Beef	2011		68	0	0	0.0	0.0	-
	2013		35	0	0	0.0	0.0	-
	2015		49	0	0	0.0	0.0	-
Dairy products	2010	bulk tank milk	57	0	0	0.0	0.0	-
	2011	cheese	76	1	0	0.0	1.3	0.0
	2014	bulk tank milk	196	1	0	0.0	0.5	0.0
Veal calves	**2010**	at farm, faeces	165	22	15	**9.1**	13.3	68.2
	**2012**	all	515	6	5	**1.0**	1.2	83.3
		at farm, faeces	217	2	2	0.9	0.9	100.0
		at slaughter, colon content	298	4	3	1.0	1.3	75.0
	**2015**	at slaughter, colon content	185	1	1	**0.5**	0.5	100.0
Veal	2012		70	4	1	1.4	5.7	25.0
Pig	**2011**	at farm, faeces, fattening pigs	859	31	13	**1.5**	3.6	41.9
	**2015**	all	730	15	11	**1.5**	2.1	73.3
		at farm, faeces, breeding pigs and piglets	512	15	11	2.1	2.9	73.3
		at slaughter, fattening pigs, colon content	218	0	0	0.0	0.0	-
Pork	2011		52	1	n.d.	-	1.9	-
	2015		43	0	0	0.0	0.0	-
**Total**	**all years**	** **	**10609**	**505**	**402**	**3.8**	**4.8**	**79.6**

n.d. not determined;—can’t be calculated

In laying hens only three *mcr-1* positive isolates were detected (among 1,809 investigated isolates). In contrast, isolates from broilers (6.7%) and chicken meat (4.3%) showed the second highest prevalence of *mcr-1* (Table 2). Different from turkeys, the prevalence of *mcr-1* positive *E*. *coli* from broiler farms was lower compared with isolates from caeca or carcass samples at the slaughterhouse of the same year. Although there is no uniform clear trend, prevalence rates at farm level decreased from 2010 to 2013 and raised slightly again in 2014. In caeca samples, collected in 2013 and 2014, prevalence rates decreased. In chicken retail meat a reduction of *mcr-1* from 8.1% and 4.9% (in 2011 and 2013) to 0.5% in 2014 was detected. The proportion of *mcr-1* in colistin resistant isolates was extremely high in broiler livestock as well as in meat samples (in retail meat 2014 there were only 2 colistin resistant isolates in total). In contrast to isolates from turkey food chain samples the proportion is decreasing over time from 100% to 88% in isolates of broiler livestock origin.

In addition to the poultry production chains breeding flocks of chickens and turkeys were included in the monitoring program for one year each. No colistin resistant isolates were detected among isolates from turkey (n = 12) or broiler breeding flocks (n = 165). Only one phenotypically colistin-resistant but *mcr-1* negative isolate was found in laying hen breeding flocks (n = 57).

The prevalence of colistin resistant and subsequently *mcr-1* harbouring *E*. *coli* among isolates from other livestock species was much lower compared to poultry samples, with the highest rate (2.4%) in veal calves (Table 2). No *mcr-1* mediated colistin resistance was detected in isolates from beef cattle and from beef and dairy products (milk and cheese). In pigs, *mcr-1* was detected in both years with a prevalence of 1.5%. As shown in Table 2, all positive isolates were from farm level samples, none from slaughter or retail. In 2015, *mcr-1* harbouring isolates were detected in samples from piglets and breeding pigs. The prevalence was slightly higher compared to the prevalence observed in 2011 in fattening pigs at farm level. In veal calves and veal at retail the prevalence of *mcr-1* in all matrices was below 1.5% in the years 2012 and 2015. In 2010, the prevalence had been considerably higher (9.1%) on farm level.

## Discussion

This report provides comprehensive data on the prevalence of *mcr-1* in representative isolates of *E*. *coli* from German livestock and food origin samples. In Germany, *mcr-1* mediated colistin resistance in *E*. *coli* occurs predominantly in the poultry production chains, whereas detection rates in bovine and porcine isolates are considerably lower. This is in contrast to reports from Asian countries, where *mcr-1* positive isolates are also frequently isolated from the pig production chain [3,18]. This may reflect differences in the antimicrobial usage patterns in pig production between Germany and the Asian countries, but data which allow comparison of usage between animal species are currently not available. In Vietnam it was described that colistin is commonly used in chicken and pig farms, and also included in commercially produced feed [30].

In Germany, the highest prevalence of *mcr-1* was found in turkeys. The higher *mcr-1* rates observed in isolates of turkey origin at farm level as compared to caeca samples at slaughter can be explained with the high frequency of use of colistin in young animals [31]. The time gap between sampling at farm and sampling at slaughter may result in a reduction of colistin resistance in the absence of selection pressure [32]. This tendency was not observed in broilers, where, due to the short life span, sampling at farm level and slaughterhouse level occur within a short period of time. This aged based reduction is observed for the prevalence of other resistance traits, too [32]. In veal calves, however, the prevalence of *mcr-1* was equally low (~1%) in isolates of faecal and caeca samples in 2012. Recent reports on *mcr-1* highlighted its presence in *E*. *coli* from pigs and cattle, but in Germany only a low prevalence was observed from these livestock origins [18,25,33].

In France *mcr-1* was also most frequently detected in isolates of turkey origin and second most from broilers as in Germany [9]. However, the prevalence of *mcr-1* in Germany was roughly twice as high as in France. There are reports about *mcr*-1 detection in samples of human, animal and food origin available from all over the world. But most publications deal with single isolates obtained by rapid screening of NGS databases whereas comprehensive epidemiologic and representative data from monitoring programs are rare [5,13,19,25]. This makes a comparison to other European countries difficult. In the European Summary Report for 2014 an EU-level prevalence of colistin resistance of 0.9% for *E*. *coli* from broilers and 7.4% in *E*. *coli* from turkeys was reported [34]. Our data indicate that the prevalence of colistin resistance in Germany is higher than the European average. A reasonable explanation for these findings might be provided by the polymyxin sales data available from the ESVAC report [27]. Relative to the extent of animal production Germany sales of polymyxins in Germany in 2013 were higher than in most other European Member States. Only Portugal, Italy and Estonia had higher sales data for polymyxins than Germany.

The *mcr-1* gene has been present in isolates from German livestock and food origin since at least 6 years with an average detection rate of 80% among colistin resistant isolates. The previous assumption that colistin resistance was limited to chromosomally mediated mechanisms is no longer relevant [1,2]. In fact, only a small proportion of colistin resistance cannot be traced back to the *mcr-1* gene. This resistance gene can be shared between strains and might also be transferred easily to other species [5,10,13]. As it was not possible to examine plasmid localisation of the gene for all 505 positive isolates this was done exemplarily with five isolates using S1-nuclease PFGE with subsequent southern blot hybridisation (data not shown). Although plasmid localisation was confirmed for these isolates, single chromosomal insertion events cannot be excluded for the remaining isolates.

In the study period, no increasing colistin resistance in human medicine can be recognized in Germany despite the high consumption in food producing animals [27]. Thanh et al. (2016) have assumed that *mcr-1* could lead to a reduced fitness of the bacteria which might be an explanation for the limited spread [15]. Actually there is a trend of decreasing prevalence of colistin resistance in general as well as *mcr-1* detection that goes along with reduced sales data of polymyxins to veterinarians in Germany. In contrast to data from Germany, colistin resistance in China increased during the last eight years [4]. Since all isolates originating from turkey and broiler breeding flocks were susceptible to colistin a vertical transmission from breeding to production flocks is probably not the main route of entry to the production flocks. However, selective isolation of colistin resistant *E*. *coli* in breeding flocks has not been attempted and very low prevalence may have gone unnoticed. Further studies highlighting the origin of the resistant strains are therefore indicated. In the production flocks, use of colistin will most likely support the spread of this type of resistance gene.

Dissemination of the *mcr-1* gene should be monitored carefully as the risk of pan resistant pathogens in human medicine has already been reported in some cases [26,35,36]. The here developed TaqMan-based real-time PCR assay provides an accurate tool for fast detection of *mcr-1*. In contrast to a recently published real-time assay the *mcr-1* gene is detected directly with a specific probe which makes it more specific than using melting point analysis [37]. This method can be used to rapidly screen the isolates collected during 2016 in the German monitoring program on zoonotic agents in poultry and to analyse if the decreasing trend will be continued for the high prevalence production chains.

## References

[1] Kempf I, Fleury MA, Drider D, Bruneau M, Sanders P, Chauvin C, et al What do we know about resistance to colistin in Enterobacteriaceae in avian and pig production in Europe? Int J Antimicrob Agents. 2013;42(5):379–83. 10.1016/j.ijantimicag.2013.06.012 . 24076115

[2] Olaitan AO, Morand S, Rolain J-M. Mechanisms of polymyxin resistance: acquired and intrinsic resistance in bacteria. Front Microbiol. 2014;5:643 10.3389/fmicb.2014.00643 . 25505462 PMC4244539

[3] Liu Y-Y, Wang Y, Walsh TR, Yi L-X, Zhang R, Spencer J, et al Emergence of plasmid-mediated colistin resistance mechanism MCR-1 in animals and human beings in China: A microbiological and molecular biological study. Lancet Infect Dis. 2015 10.1016/S1473-3099(15)00424-7 26603172

[4] Shen Z, Wang Y, Shen Y, Shen J, Wu C. Early emergence of *mcr-1* in *Escherichia coli* from food-producing animals. Lancet Infect Dis. 2016;16(3):293 10.1016/S1473-3099(16)00061-X 26973308

[5] Kuo S-C, Huang W-C, Wang H-Y, Shiau Y-R, Cheng M-F, Lauderdale T-L. Colistin resistance gene *mcr-1* in *Escherichia coli* isolates from humans and retail meats, Taiwan. J Antimicrob Chemother. 2016 10.1093/jac/dkw122 . 27076107

[6] Grami R, Mansour W, Mehri W, Bouallègue O, Boujaâfar N, Madec J-Y, et al Impact of food animal trade on the spread of *mcr-1-*mediated colistin resistance, Tunisia, July 2015. Euro Surveill. 2016;21(8):30144 10.2807/1560-7917.ES.2016.21.8.30144 . 26940999

[7] Olaitan AO, Chabou S, Okdah L, Morand S, Rolain J-M. Dissemination of the *mcr-1* colistin resistance gene. Lancet Infect Dis. 2016;16(2):147 10.1016/S1473-3099(15)00540-X 26711360

[8] Khalifa HO, Ahmed AM, Oreiby AF, Eid AM, Shimamoto T, Shimamoto T. Characterisation of the plasmid-mediated colistin resistance gene *mcr-1* in *Escherichia coli* isolated from animals in Egypt. Int J Antimicrob Agents. 2016 10.1016/j.ijantimicag.2016.02.011 . 27112794

[9] Perrin-Guyomard A, Bruneau M, Houée P, Deleurme K, Legrandois P, Poirier C, et al Prevalence of *mcr-1* in commensal *Escherichia coli* from French livestock, 2007 to 2014. Euro Surveill. 2016;21(6):30135 Available from: http://www.eurosurveillance.org/ViewArticle.aspx?ArticleId=21380. 10.2807/1560-7917.ES.2016.21.6.30135 26898350

[10] Quesada A, Ugarte-Ruiz M, Iglesias MR, Porrero MC, Martínez R, Florez-Cuadrado D, et al Detection of plasmid mediated colistin resistance (MCR-1) in *Escherichia coli* and *Salmonella enterica* isolated from poultry and swine in Spain. Res Vet Sci. 2016;105:134–5. 10.1016/j.rvsc.2016.02.003 . 27033921

[11] Rapoport M, Faccone D, Pasteran F, Ceriana P, Albornoz E, Petroni A, et al First descirption of m*cr-1*-mediated colistin resistance in human infections caused by *Escherichia coli* in Latin America. Antimicrob Agents Chemother. 2016 10.1128/AAC.00573-16 . 27090181 PMC4914662

[12] Mulvey MR, Mataseje LF, Robertson J, Nash JHE, Boerlin P, Toye B, et al Dissemination of the *mcr-1* colistin resistance gene. Lancet Infect Dis. 2016;16(3):289–90. 10.1016/S1473-3099(16)00067-0 26973304

[13] Doumith M, Godbole G, Ashton P, Larkin L, Dallman T, Day M, et al Detection of the plasmid-mediated *mcr-1* gene conferring colistin resistance in human and food isolates of *Salmonella enterica* and *Escherichia coli* in England and Wales. J Antimicrob Chemother. 2016 10.1093/jac/dkw093 . 27090630

[14] Zeng K-J, Doi Y, Patil S, Huang X, Tian G-B. Emergence of plasmid-mediated *mcr-1* gene in colistin-resistant *Enterobacter aerogenes* and *Enterobacter cloacae*. Antimicrob Agents Chemother. 2016 10.1128/AAC.00345-16 . in press. 26976876 PMC4879368

[15] Thanh DP, Tuyen HT, Nguyen Thi Nguyen T, The HC, Wick RR, Thwaites G, et al Inducible colistin resistance via a disrupted plasmid-borne *mcr-1* gene in a 2008 Vietnamese *Shigella sonnei* isolate. J. Antimicrob Chemother. 2016 10.1093/jac/dkw173 PMC4954929 27246235

[16] Ye H, Li Y, Li Z, Gao R, Zhang H, Wen R, et al Diversified *mcr-1*-harbouring plasmid reservoirs confer resistance to colistin in human gut microbiota. mBio. 2016;7(2):16 10.1128/mBio.00177-16 . 27048797 PMC4817250

[17] Zurfuh K, Poirel L, Nordmann P, Nüesch-Inderbinen M, Hächler H, Stephan R. Occurrence of the plasmid-borne *mcr-1* Colistin resistance gene in extended-spectrum-β-lactamase-producing Enterobacteriaceae in river water and imported vegetable samples in Switzerland. Antimicrob Agents Chemother. 2016;60(4):2594–5. 10.1128/AAC.00066-16 . 26883696 PMC4808203

[18] Malhotra-Kumar S, Xavier BB, Das AJ, Lammens C, Hoang HTT, Pham NT, et al Colistin-resistant *Escherichia coli* harbouring *mcr-1* isolated from food animals in Hanoi, Vietnam: A microbiological and molecular biological study. Lancet Infect Dis. 2016;16(3):286–7. 10.1016/S1473-3099(16)00014-1 26774248

[19] Hasman H, Hammerum AM, Hansen F, Hendriksen RS, Olesen B, Agersø Y, et al Detection of *mcr-1* encoding plasmid-mediated colistin-resistant *Escherichia coli* isolates from human bloodstream infection and imported chicken meat, Denmark 2015. Euro Surveill. 2015;20(49):30085 10.2807/1560-7917.ES.2015.20.49.30085 . 26676364

[20] Kluytmans–van den Bergh Marjolein F, Huizinga P, Bonten MJ, Bos M, Bruyne K de, Friedrich AW, et al Presence of *mcr-1* -positive Enterobacteriaceae in retail chicken meat but not in humans in the Netherlands since 2009. Euro Surveill. 2016;21(9):30149 10.2807/1560-7917.ES.2016.21.9.30149 26967540

[21] Cannatelli A, Giani T, Antonelli A, Principe L, Luzzaro F, Rossolini GM. First Detection of the *mcr-1* Colistin Resistance Gene in *Escherichia coli* in Italy. Antimicrob Agents Chemother. 2016;60(5):3257–8. 10.1128/AAC.00246-16 . 26976865 PMC4862502

[22] Prim N, Rivera A, Rodríguez-Navarro J, Español M, Turbau M, Coll P, et al Detection of *mcr-1* colistin resistance gene in polyclonal *Escherichia coli* isolates in Barcelona, Spain, 2012 to 2015. Euro Surveill. 2016;21(13):30183 10.2807/1560-7917.ES.2016.21.13.30183 . 27055477

[23] Skov RL, Monnet DL. Plasmid-mediated colistin resistance (*mcr-1* gene): three months later, the story unfolds. Euro Surveill. 2016;21(9):30155 10.2807/1560-7917.ES.2016.21.9.30155 . 26967914

[24] Poirel L, Kieffer N, Liassine N, Thanh D, Nordmann P. Plasmid-mediated carbapenem and colistin resistance in a clinical isolate of *Escherichia coli*. Lancet Infect Dis. 2016;16(3):281 10.1016/S1473-3099(16)00006-2 26774246

[25] Falgenhauer L, Waezsada S-E, Yao Y, Imirzalioglu C, Käsbohrer A, Roesler U, et al Colistin resistance gene *mcr-1* in extended-spectrum β-lactamase-producing and carbapenemase-producing Gram-negative bacteria in Germany. Lancet Infect Dis. 2016;16(3):282–3. 10.1016/S1473-3099(16)00009-8 26774242

[26] Yao X, Doi Y, Zeng L, Lv L, Liu J-H. Carbapenem-resistant and colistin-resistant *Escherichia coli* co-producing NDM-9 and MCR-1. Lancet Infect Dis. 2016;16(3)288–9 10.1016/S1473-3099(16)00057-8 26842777

[27] European Medicines Agency. Sales of veterinary antimicrobial agents in 26 EU/EEA countries in 2013. Fifth ESVAC report.; EMA/387934/2015.

[28] Roschanski N, Fischer J, Guerra B, Roesler U. Development of a multiplex real-time PCR for the rapid detection of the predominant beta-lactamase genes CTX-M, SHV, TEM and CIT-type AmpCs in Enterobacteriaceae. PLoS ONE. 2014;9(7):e100956 10.1371/journal.pone.0100956 . 25033234 PMC4102473

[29] Kaesbohrer A, Schroeter A, Tenhagen BA, Alt K, Guerra B, Appel B. Emerging antimicrobial resistance in commensal Escherichia coli with public health relevance. Zoonoses Public Health. 2012;59 Suppl 2:158–65. 10.1111/j.1863-2378.2011.01451.x . 22958260

[30] Nguyen NT, Nguyen HM, Nguyen CV, Nguyen TV, Nguyen MT, Thai HQ, et al Use of colistin and other critical antimicrobials on pig and chicken farms in southern Vietnam and its association with resistance in commensal *Escherichia coli*. Appl Environ Microbiol. 2016 10.1128/AEM.00337-16 . in press. 27084016 PMC4907207

[31] Ministerium für Klimaschutz, Umwelt, Landwirtschaft, Natur-und Verbraucherschutz des Landes Nordrhein-Westfalen. https://www.umwelt.nrw.de/pressebereich/pressemitteilung/news/2014-11-25-minister-remmel-einsatz-von-antibiotika-in-der-intensivtierhaltung-ist-alarmierend/.

[32] Niedersächsisches Landesamt für Verbraucherschutz und Lebensmittelsicherheit, Niedersächsisches Ministerium für Ernährung, Landwirtschaft, Verbraucherschutz und Landesentwicklung. Bericht über den Antibiotikaeinsatz in der landwirtschaftlichen Nutztierhaltung in Niedersachsen; 2011.

[33] Haenni M, Poirel L, Kieffer N, Châtre P, Saras E, Métayer V, et al Co-occurrence of extended spectrum β lactamase and MCR-1 encoding genes on plasmids. Lancet Infect Dis. 2016;16(3):281–2. 10.1016/S1473-3099(16)00007-4 26774244

[34] European Food Safety Authority, European Centre for Disease Prevention and Control. The European Union summary report on antimicrobial resistance in zoonotic and indicator bacteria from humans, animals and food in 2014. EFSA Journal. 2016;14(2):4380 10.2903/j.efsa.2016.4380 PMC7009656 32625816

[35] Li A, Yang Y, Miao M, Chavda KD, Mediavilla JR, Xie X, et al Complete sequences of *mcr-1*-harboring plasmids from extended spectrum β-lactamase (ESBL)- and carbapenemase-producing Enterobacteriaceae (CPE). Antimicrob Agents Chemother. 2016;60(7):4351–4. 10.1128/AAC.00550-16 . 27090180 PMC4914624

[36] Du H, Chen L, Tang Y-W, Kreiswirth BN. Emergence of the *mcr-1* colistin resistance gene in carbapenem-resistant Enterobacteriaceae. Lancet Infect Dis. 2016;16(3):287–8. 10.1016/S1473-3099(16)00056-6 26842776

[37] Bontron S, Poirel L, Nordmann P. Real-time PCR for detection of plasmid-mediated polymyxin resistance (*mcr-1*) from cultured bacteria and stools. J Antimicrob Chemother. 2016 10.1093/jac/dkw139 . 27121402

